# Pro-fibrogenic and adipogenic aspects of chronic muscle degeneration are contributed by distinct stromal cell subpopulations

**DOI:** 10.1371/journal.pone.0288800

**Published:** 2023-07-18

**Authors:** Cansu Özdemir, Duygu Akçay, Diğdem Yöyen-Ermiş, Ekim Zihni Taşkıran, Rana Soylu-Kucharz, Güneş Esendağlı, Yusuf Çetin Kocaefe

**Affiliations:** 1 Department of Stem Cell Sciences, Graduate School of Health Sciences, Hacettepe University, Ankara, Turkey; 2 Center for Stem Cell Research and Development, Hacettepe University, Ankara, Turkey; 3 Department of Medical Biology, School of Medicine, Hacettepe University, Ankara, Turkey; 4 Department of Basic Oncology, Cancer Institute, Hacettepe University, Ankara, Turkey; 5 Department of Immunology, School of Medicine, Uludağ University, Bursa, Turkey; 6 Department of Medical Genetics, School of Medicine, Hacettepe University, Ankara, Turkey; Keio University - Shinanomachi Campus: Keio Gijuku Daigaku - Shinanomachi Campus, JAPAN

## Abstract

Chronic skeletal muscle degeneration is characterized by fiber atrophy accompanied by deposition of extracellular matrix (ECM) components and fatty infiltration. Excessive accumulation of ECM leads to fibrosis via the contribution of fibro-adipogenic precursors (FAPs). Fibrosis also accompanies disuse atrophy and sarcopenia without significant inflammation. The present study aimed to comparatively analyze heterogeneous population of FAPs during acute injury and immobilization (tenotomy and denervation). The comparative analysis was accomplished based on the following 3 stromal cell subpopulations: i) CD140a^(+)^/Sca1^(+)^; ii) CD140a^(+)^/Sca1^(–)^; iii) CD140a^(–)^/Sca1^(+)^. RNASeq analysis was employed to characterize and compare their quiescent and activated states. Whereas CD140a^(-)^/Sca1^(+)^ was the most predominant activated subpopulation in tenotomy, denervation stimulated the CD140a^(+)^/Sca1^(+)^ subpopulation. Immobilization models lacked myofiber damage and exhibited a minute increase in CD45^(+)^ cells, as compared to acute injury. Transcriptome analysis showed common and discordant regulation of ECM components, without profound proliferative activation. Herein, we suggest unique surface markers for further identification of the investigated cell subpopulations. FAP subpopulations show similar activation kinetics in an inflammatory environment but the present findings highlight the fact that inflammation may not be a prerequisite for FAP activation. Delayed proliferation kinetics indicate that signals beyond inflammation might trigger FAP activation, leading to irreversible stromal changes.

## Introduction

Skeletal muscle injury is repaired by the concerted activation of tissue resident muscle precursors and stromal cells; however, recurrent cycles of injury and repair in cases of dystrophies or myopathies result in chronic skeletal muscle degeneration characterized by fiber atrophy, deposition of extracellular matrix (ECM) components, and fatty infiltration [1]. ECM is not only required for tissue development and support, but is also essential for locomotor activity. Excessive accumulation and thickening of ECM components leads to irreversible tissue architectural changes, along with an increase in stiffness; such deterioration is defined as fibrosis. Fibrosis impairs skeletal muscle function, maintenance, and repair in several ways. ECM deposition deteriorates the niche of skeletal muscle precursors, resulting in impaired satellite cell activation and migration [1, 2]. Excessive thickening of ECM also impairs the release and transduction of regenerative paracrine signals [1, 3]. Furthermore, increased stiffness of fibrotic muscle negatively impacts kinetic capacity, triggering myofiber atrophy [1]. Overall, chronic progressive structural changes irreversibly impair muscle regenerative capacity and function; therefore, effective treatment of muscle degeneration cannot be achieved without prevention of fibrosis [4, 5]. The fundamentals of signaling and the cellular origins of fibrosis must be better understood in order to develop preventive strategies.

Multiple stromal cells of skeletal muscle that contribute to injury repair as well as degeneration have been characterized [6, 7]. Among them, fibro-adipogenic precursors (FAPs) are the major mesenchymal stromal cell population contributing to injury repair and fibrosis [8, 9]. FAPs are well known for their activation associated with acute injury repair via 2 main inflammatory cytokines: TNFα and TGFβ [10]. Whereas FAPs are capable of differentiating into osteogenic, adipogenic, and myo-fibroblastic lineages, they do not fuse to form skeletal muscle fibers [9, 11]. FAPs are a heterogeneous population lacking precise surface makers, but immunophenotyping using CD140a^(+)^, Sca1^(+)^, CD31^(–)^, CD45^(–)^, and CD11b^(–)^ was shown to be distinctive [8].

In the case of acute injury, FAPs are known to support the repair process by secreting growth factors such as IGF1, and then disappear following repair via apoptosis via TNFα signaling [12]; however, in a chronic inflammatory environment, sustained TGFβ signaling causes FAPs to maintain their proliferative state and induces their differentiation into myofibroblasts that express alpha-smooth muscle actin (*Acta2*) and collagen type-I, along with several ECM components, contributing to fibrosis [12]. Chronic inflammation is the hallmark of dystrophies characterized by chronic muscle degeneration and fibrosis [13, 14]; however, fibrosis also accompanies disuse atrophy and sarcopenia, in the absence of significant inflammation [15, 16]. Nonetheless, the cellular components and signaling cascades that contribute to fibrosis are not completely understood.

The present study aimed to determine the activation state of stromal cell subpopulations (presumably FAPs) in 2 distinct models that mimic diverse manifestations of chronic muscle degeneration. Tenotomy immobilization and denervation induce endomysial fibrosis and fatty infiltration, respectively, without significant inflammation. Herein we show that activation of distinct stromal cell subpopulations in dissimilar immobilization models leads to predominant adipogenic or fibrogenic differentiation, depending on the mode of immobilization. Transcriptome analysis of these distinct subpopulations shows evidence of diverse activation mechanisms in different models of tissue degeneration. The present findings suggest there are novel candidate surface markers for stromal cells with potential implications in clinical samples.

## Materials and methods

### Animals and procedures

**Fig 1:**
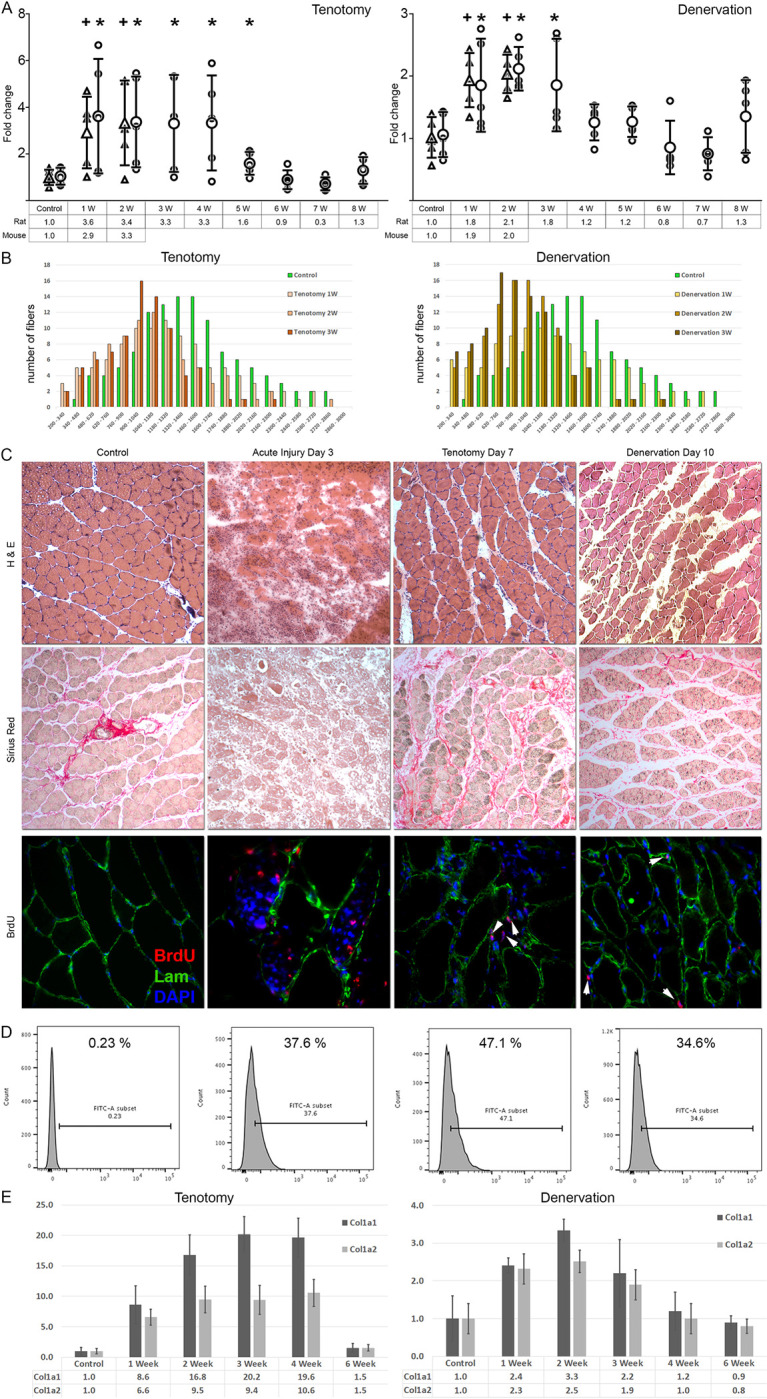
Hind limb mononuclear cells from time-course tenotomy and denervation muscles were isolated, enumerated, and normalized to the contralateral muscles for the indicated time points in rats and mice (circles for rats and triangles for mice, n = 5, A). Denervation induced less of an increase in the mononuclear cell population, lasting until week 3. Compared to controls, statistically significant increase in the mononuclear cell population was evident from week 1 to week 4 in tenotomy (+ and * symbols indicate statistical significance of p<0.05 for mice and rats respectively). Atrophy kinetics of the tenotomized and denervated muscles are investigated by fiber cross-sectional area measurements (x-axis values are in μm^2^, B). The representative images of the time-points selected for further evaluation in this study are presented in panel C. Fibrotic changes in tenotomy samples were evident on d 7. Sirius red staining showed marked deposition of collagens, along with atrophy. Denervation resulted in more severe atrophy without collagen deposition on d 10 (Sirius red staining in C). BrdU staining showed extensive positive nuclei in muscles subjected to acute injury on d 3, but limited positivity in both denervation and tenotomy immobilized muscles. The positive nuclei were exclusively limited to the stromal compartment outside the laminin demarcated area (arrowheads, red staining, C). BrdU incorporation into the stromal mononuclear cells are investigated via flow cytometry (D) and the time-course expression of Col1a1 and Col1a2 is documented to show collagen deposition in models (E).

All methods were performed in accordance with the relevant guidelines and regulations. All procedures were performed according to an institution-approved protocol under strict biological containment. The study was approved by the Hacettepe University Ethics Committee for Animal Experimentation (approval decisions: 2014/57-04, 2007/11-1 and 2009/30-4). All methods utilized in this study were in accordance and full compliance with ARRIVE guidelines. Technical and conceptual validation of previous observations on chronic immobilization models in rats are performed on mice (n = 5 for each group). (Mononuclear cell counts and atrophy kinetics are summarized in Fig 1A and 1B). Unpaired Student’s t-test with a cut-off of p<0.05 was used to determine the significance of group-wise variations of mononuclear cell counts (compared to controls) and sub-populations. Atrophy kinetics of the immobilization models were evaluated via fiber cross-sectional area measurements as described previously [17].

The study included 8-12-week-old Swiss white mice weighing 20 ± 5 g. Interventions were performed on the right legs and contralateral leg muscles were designated as controls. BrdU was administered daily via intraperitoneal injections of 50 mg kg^–1^ (Sigma B5002) in 0.9% saline. Following ketamine/xylazine anesthesia (90/10 mg kg^–1^ IP) and local disinfection, acute muscle injury was generated via cardiotoxin injection (20 uM in sterile PBS, Sigma C5759) into the right hind limb muscles (n = 5). Chronic immobilization models were generated with tendon release tenotomy and denervation via sciatic nerve resection, as described previously [18, 19]. The tibialis anterior muscle was resected under general anesthesia 3 d and 7 d after intervention (n = 10 for each time point). All resected muscles were immediately processed as described below and the animals were sacrificed by overdose anesthesia.

### Isolation of mononuclear cells

Resected muscles were weighed and minced. Enzymatic digestion was performed under continuous rotary mixing for 15 min at 37°C in 2 mL of 1% collagenase/dispase solution (Collagenase B Roche 11088831001, Dispase-II Roche D4693) and 1 μg of DNaseI (Promega: M6101) for each gram of muscle. Undigested tissue clumps were precipitated at 100 G for 3 min and supernatant cells were filtered through 100-μm mesh. The filtrate was centrifuged at 300 G for 5 min at 4°C. Tissue clumps and filter-retained tissues were further digested until total dissociation. The mononuclear cell pellet was filtered through 40-μm mesh and subjected to gradient centrifugation to eliminate cellular debris (Ficoll Sigma F4375). Cells were incubated in PBS solution supplemented with 2% FBS in a CO_2_ incubator at 37°C for 8 h of surface marker recovery under mild continuous stirring conditions (20 RPM).

### Flow cytometry

Immunophenotyping of mononuclear cells was performed using the following monoclonal antibodies: anti-mouse PDGFRα-APC (CD140a, BD-562777); Ly6A/E-PE (Sca1, BD-553108); CD11b-FITC (BD-561688); CD31-FITC (BD-558738); CD45-FITC (BD-561088). The percentage of positive cells was determined in consideration of the following isotype matched antibodies: IgG2a-κ (APC: BD553932, PE: BD553930); IgG2b-κ-FITC (BD553988); IgG2a- κ-FITC (BD553929). An Anti-BrdU Flow Kit (Sigma B5002) and BrdU-FITC Flow Kit (BD Pharmingen 559619) were used to assess BrdU positivity. Sca1^(+)^, CD140a^(–)^, CD31^(–)^, CD45^(–)^, and CD11b^(–)^ cells were purified using fluorescent-activated cell sorting (FACS) with a purity ≥97%. Staining was performed using standard techniques according to the manufacturer’s instructions. Flow cytometry and cell sorting were performed using a FACS Aria 2 (BD Biosciences, San Jose, CA).

### Cell culture

Sorted cells (Sca1^(+)^, CD140a^(–)^, CD31^(–)^, CD45^(–)^, CD11b^(–)^) were seeded at 10^4^ cells cm^–2^ and cultured on Matrigel (Corning 356235)-coated 6-well plates in high-glucose DMEM (Thermo 11965092) supplemented with 5 ng mL^–1^ of bFGF (Sigma F3685), FBS 20% (GE SH30071), 2 mM of L-glutamine (Sigma G3126), Amphotericin B 1% (Sigma A2942), and penicillin/streptomycin 1% (Biochrome A2210). After stromal cell expansion, adipogenic differentiation was induced via reducing FBS to 5% and replacing bFGF with 0.5 mM of IBMX (Sigma I5879), 0.25 μM of dexamethasone (Sigma D4902), and 10 μg mL^–1^ of insulin (Sigma I1507), as described previously [20].

### Histochemistry, immunostaining and RT-PCR

Skeletal muscle tissues were collected and blocked within OCT compound, and sectioned to 8-μm thickness. Standard hematoxylin & eosin and sirius red staining procedures were employed [21, 22]. Tissue sections were co-stained with Laminin (Sigma SAB4200719, 1:100 dilution) and BrdU (Abcam 6326, working dilution 1:100) antibodies, and appropriate fluorescent-labelled secondary antibodies (Abcam ab150167 and ab150117, working dilution 1:1000). Then, 1 μg mL^–1^ of DAPI (Sigma-Aldrich, D9542) was applied to cover slips as a nuclear stain. Immunofluorescent images were captured using a Leica DMIL microscope equipped with a DFC 320 camera.

At the time of tissue harvesting, 30 to 50 mg of midbelly muscle sample was resected and was subjected to total RNA extraction and cDNA synthesis as described before [18]. Quantitative real-time expression of Col1a1 and Col1a2 genes were assessed using SYBR-green method, relative expression of the two transcripts were achieved using normalization strategy described elsewhere [23].

### RNA isolation and transcriptome analysis

Total RNA was isolated from freshly sorted stromal cell populations using Trizol reagent (Invitrogen 15596–026), according to the manufacturer’s instructions. RNA quantitation was performed using a Qubit™ RNA HS Assay Kit (Invitrogen Q32852). In total, 20–100 ng of total RNA was employed to prepare the mRNA library using a QuantSeq 3’mRNA-Seq Library Prep Kit (Lexogen), according to the manufacturer’s standard protocol. Libraries were quantitated using a Qubit dsDNA High-Sensitivity Quantitation Kit (Invitrogen Q32854). Next, 100 pM of library dilutions were prepared, barcoded, and pooled via emulsion PCR using a PI Hi-Q OT2 200 device (Thermo Fisher Scientific) and Ion One Touch 2 device (Thermo Fisher Scientific). After clonal DNA amplification, libraries were purified from magnetic beads using an Ion One Touch ES device. Sequencing was accomplished using Ion PI Hi-Q Sequencing 200 reagents (Thermo Fisher Scientific A26434) on an Ion PI Chip v3 (Thermo Fisher A26771) and Ion Proton system (Thermo Fisher 4476610).

Generated uBAM files were initially de-multiplexed and trimmed using FastX-Toolkit (Gordon A, Hannon G. Fastx-toolkit. FASTQ/A short-reads pre-processing tools 2010. Available from: http://hannonlab.cshl.edu/fastx_toolkit/). Reads were aligned to the Ensembl mouse genome (GRCm38/mm10 release 90) using STAR [24] and BRB-seqtools v.1.2. Read counts were further normalized using DESeq2 for R [25]. Normalized gene counts were annotated, processed, and analyzed using BRB-arraytools v.4.6 [26]. An initial filtering strategy was employed to exclude low/non-expressing genes, with the following cut-off threshold: the 80th percentile of expression values was <8 and a minimum fold change of variation for each gene exhibiting a fold change deviation <1.5-fold of the median expression value to generate gene lists. A “Class Comparison” is employed to perform a t-test for each gene using the normalized log-ratios using random variance to identify genes that are differentially expressed among groups.

## Results

### Acute injury and immobilization models activate distinct stromal cell subpopulations

Denervation and tenotomy are distinct immobilization models that predominantly demonstrate two dissimilar features of chronic skeletal muscle degeneration—fatty infiltration and fibrosis, respectively. In order to observe the activation kinetics of mononuclear cells, a time-course experiment was employed using the two immobilization models. We have previously characterized and reported investigations on whole muscle tissue regarding tenotomy immobilization on rats elsewhere [18, 19]. Immunophenotyping materials required for the characterization of FAPs are not available for the rat. Thus, we adapted and standardized both immobilization models reported herein in mice (Fig 1A). Interventions were performed on unilateral hindlimbs, and then mononuclear cells were isolated, enumerated, and normalized to the contralateral limb. Hindlimb (gastrocnemius and soleus) muscles were used for acute injury. Tibialis anterior muscle was used for the two immobilization models. Statistically significant time-points exhibiting the maximum increase in stromal cell subpopulations preceding chronic architectural changes were selected (d 10 for denervation, and d 3 and d 7 for tenotomy). The animals received daily bromodeoxyuridine (BrdU) injections to trace the proliferating cells. The atrophy kinetics of the two models are documented using fiber cross-sectional area measurements (Fig 1B).

In the case of acute injury, skeletal muscle samples obtained on day 3 were selected for characterization based on the fact that FAPs were previously reported to reach their peak activation on day 3 [10]. Several BrdU-positive nuclei, both within the endomysial and perimysial compartments were visible following acute injury (Fig 1C). In contrast, a very limited number of cells were observed to be BrdU-positive in the immobilization models. These BrdU-positive cells resided exclusively outside the laminin-demarcated borders and, thus, were of the stromal population. Several muscle sample sections from the immobilization models were investigated for BrdU positivity, but positive myonuclei were not observed. Typical representative histograms showing BrdU positivity of the mononuclear cells are shown in Fig 1D.

In principle, the stromal fraction consists of progenitors, endothelial cells, and stromal connective tissue cells, as well as tissue-residing macrophages and other cells of hematopoietic origin. Stromal degenerative changes in skeletal muscle were previously associated with a certain cell population that was conceptually referred as fibro-adipogenic precursors (FAPs). Various marker panels for identifying FAPs have been reported [8, 27]. The present study aimed to further characterize these cell populations using a consensus panel consisting of a negative selection based on CD45, CD31, CD11b (excluding hematopoietic and endothelial cells), and a positive and differential selection based on CD140a (Pdgfra) and Sca1 (Ly6A/E). Acute injury was used as a positive control for stromal cell activation.

**Fig 2:**
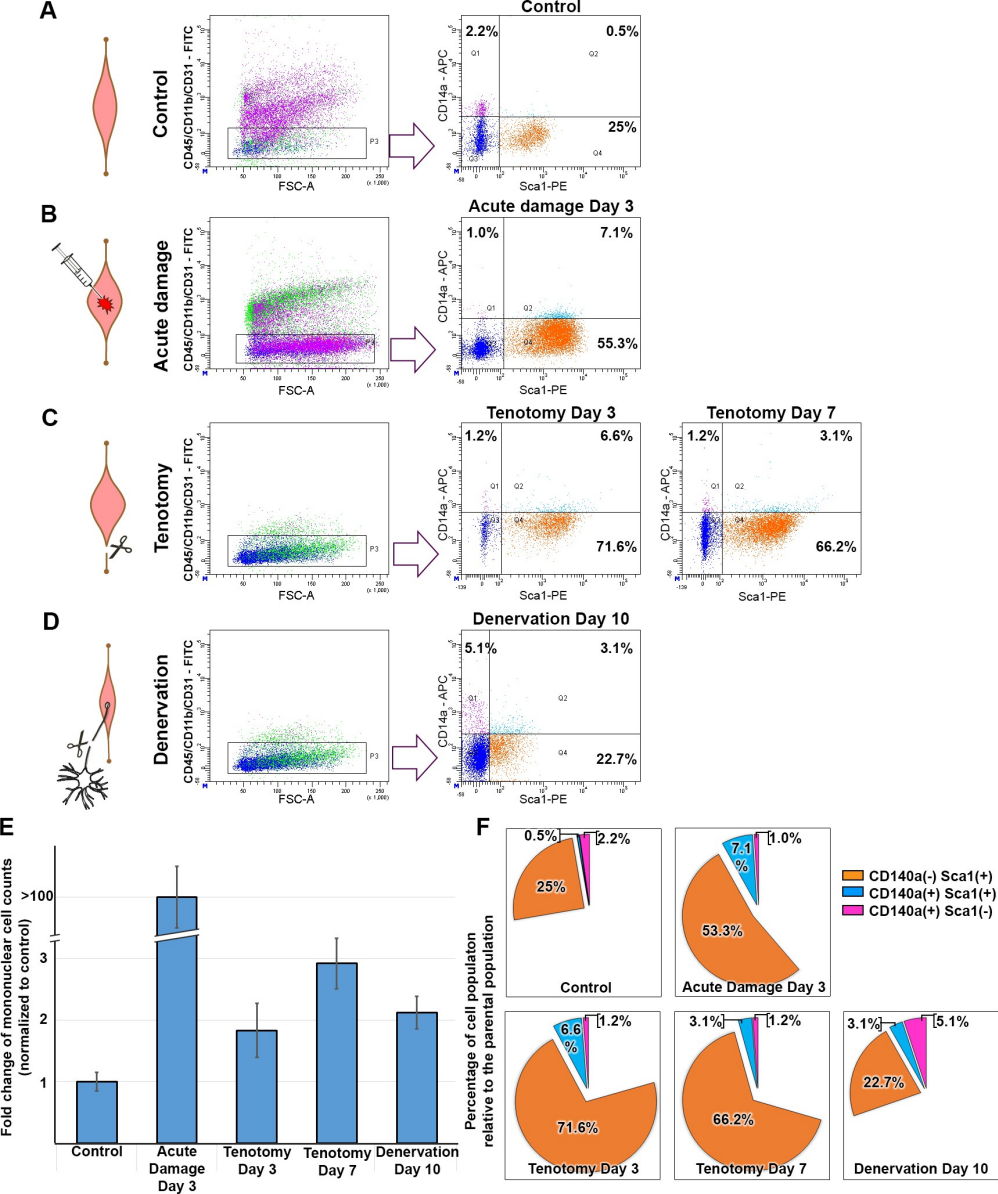
Stromal cell subpopulations following acute injury (d 3), tenotomy (d 3 and d 7), and denervation (d 10) were selected for CD45, CD31, and CD11b negativity (gating strategy) and further analyzed for CD140a^(+)^/Sca1^(−)^, CD140a^(−)^/Sca1^(+)^, and CD140a^(+)^/Sca1^(+)^ subpopulations compared to the control (A). Acute injury resulted in activation of the CD140a^(−)^/Sca1^(+)^ and CD140a^(+)^/Sca1^(+)^ subpopulations (B). Likewise, tenotomy resulted in activation of the CD140a^(−)^/Sca1^(+)^ and CD140a^(+)^/Sca1^(+)^ subpopulations on d 3 and d 7 (C), whereas denervation resulted in activation of the CD140a^(+)^/Sca1^(−)^ and CD140a^(+)^/Sca1^(+)^ subpopulations (D). Variations of mononuclear cell numbers in studied models and time points are presented (E). Relative representative abundance of the investigated subpopulations is presented as a pie chart (F). All experiments are conducted on freshly isolated cells (n = 5 for acute injury, n = 10 for immobilization models) and independently triplicated. Typical (median) flow cytometry panels are shown.

Acute injury (cardiotoxin n = 5), denervation (sciatic nerve n = 10) and tenotomy (tibialis anterior n = 10) models were employed in mice, and mononuclear cells were isolated. Contralateral muscles were untreated and employed as controls. Mononuclear cells from acute injury day 3, denervation day 10, and tenotomy days 3 and 7 were isolated and immunophenotyped using the above-mentioned markers (Fig 2E). A gating strategy was used to exclude CD45, CD31, and CD11b positivity (Fig 2). The negative population was further characterized for CD140a^(+)^/Sca1^(–)^, CD140a^(–)^/Sca1^(+)^, and double positive (CD140a^(+)^/Sca1^(+)^) subpopulations. Typical representative flow cytometry panels are shown in Fig 2A–2D. Relative percentages of the investigated subpopulations are represented in Fig 2F.

Percentages of the subpopulations are presented in Table 1 (averages of 3 independent biological replicates). Acute injury caused a 3.5-fold increase in the CD140a^(+)^/Sca1^(+)^ subpopulation (2.1% in controls and 7.1% in acute injury). In addition, the CD140a^(–)^/Sca1^(+)^ subpopulation increased 2-fold (27.3% to 52.4%). Likewise the acute injury, tenotomy immobilization induced a steady, more than 2-fold increase in CD140a^(-)^/Sca1^(+)^ subpopulation in both d3 and d7 (25% in controls vs 71% and 66% on d 3 and 7 respectively). The CD140a^(+)^/Sca1^(+)^ subpopulation also showed more than 2-fold increase on d 3 following tenotomy but did not meet statistical significance. Denervation, did not exhibit significant change on CD140a^(–)^/Sca1^(+)^ subpopulation, unlike in the acute injury or tenotomy models. CD140a^(+)^/Sca1^(–)^ subpopulation exhibited a 2-fold increase (2.3 in control and 5.4 in denervation). CD140a^(-)^/Sca1^(+)^ subpopulations exhibited 2-fold upregulation in the acute injury and tenotomy models, whereas the CD140a^(+)^/Sca1^(–)^ subpopulation exhibited an increase in the denervation immobilization model. These findings clearly show that distinct stromal cell subpopulations are activated in different immobilization models.

**Table 1:** Percentage abundance (average of triplicated experiments) of the investigated subpopulations.

	Control	Acute Damage Day 3	Tenotomy Day 3	Tenotomy Day 7	Denervation Day 10
CD140a^(-)^ Sca1^(+)^	27.3 (±5.3)	52.4 (±3.1) *	66.7 (±4.6) *	59.7 (±6.7) *	22 (±3.4)
CD140a^(+)^ Sca1^(+)^	2.1 (±3.1)	7.1 (±0.4) *	6.3 (±0.5)	3.1 (±0.4)	3.0 (±0.2)
CD140a^(+)^ Sca1^(-)^	2.3 (±0.8)	1.1 (±0.2)	1.4 (±0.2)	1.2 (±0.1)	5.4 (±1.1) *

(*) Statistically significant variances compared to controls (p<0.05).

### Limited inflammation and stromal cell activation in immobilization models

**Fig 3:**
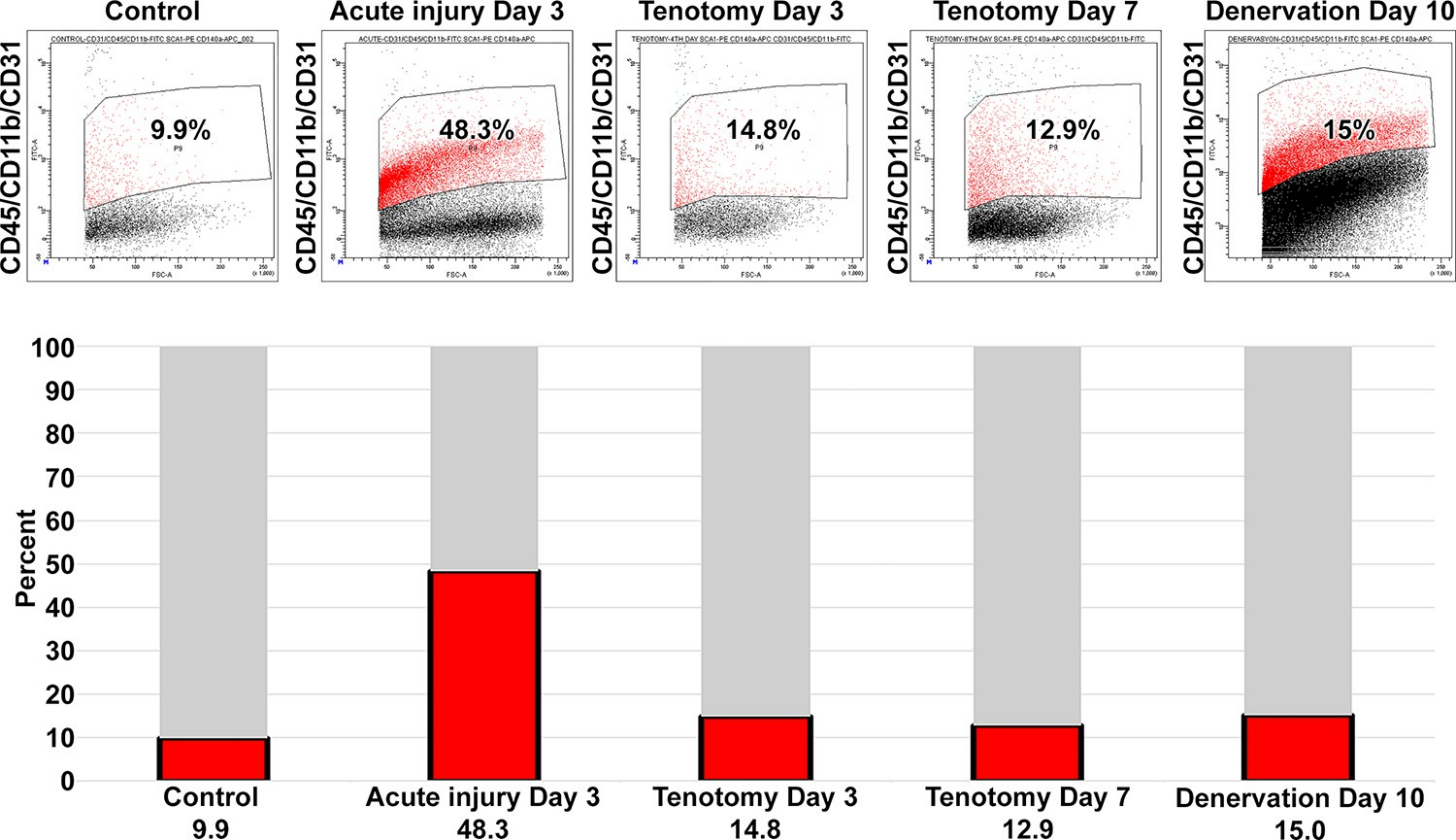
CD45/CD11b/CD31^(+)^ cell populations were enumerated in the studied models using flow cytometry (pooled muscle samples: N = 10). Typical cytometry panels are presented and inflammatory cell percentages are provided as a fraction of total mononuclear cells. On d 3 of acute injury the maximum percentage of inflammatory cells among stromal cells was observed.

We further analyzed the dynamics of CD45/CD11b/CD31 cell populations in immobilization models. In the control muscles the percentage of these cells was 9.9% (Fig 3). On day 3 of acute injury this percentage increased to 48.3%, along with inflammation. This increase in endothelial and/or inflammatory cells was much more limited in the immobilization models. Following tenotomy, the CD45/CD11b/CD31(+) cell percentage was 14.8% and 12.9% on d 3 and d 7, respectively (Fig 3). Likewise, the percentage was 15% on day 10 of denervation. These data indicate that the increase in inflammatory and/or endothelial cells observed in the acute injury model was more limited in the immobilization models (or there was a concomitant decrease in the stromal cell subpopulation that was excluded in the analysis, which is an unlikely scenario).

In order to further elucidate the importance of this observation, we enumerated BrdU positivity in all cells exhibiting stromal cell markers. In control muscles, BrdU positivity was observed in ≈2.8% of all stromal cells. The distribution of BrdU-positive cells is presented in S1 Fig. In control muscles the CD140a^(+)^/Sca1^(+)^ subpopulation exhibited the highest BrdU incorporation (8.2%), whereas the CD140a^(+)^/Sca1^(–)^ subpopulation exhibited the lowest (2.8%). These findings indicate that there is limited physiological turnover of these cells in control muscles, as mentioned above (S1 Fig).

Following acute injury, the greatest increase in of BrdU positivity was observed in the CD140a^(+)^/Sca1^(–)^ subpopulation (S1 Fig), which increased from 2.8% to 68.1%. Both the CD140a^(+)^/Sca1^(+)^) and CD140a^(–)^/Sca1^(+)^ subpopulations exhibited an 8-fold increase in BrdU positivity. The immobilization models exhibited a much more limited increase in BrdU incorporation, as compared to acute injury, but were similar to each other. All subpopulations showed an increase ranging from 10% in the CD140a^(+)^/Sca1^(–)^ subpopulation up to 33.1% in the CD140a^(+)^/Sca1^(+)^ subpopulation (S1 Fig). These data support the above-mentioned findings of limited proliferative activation of the stromal cell subpopulations in the immobilization models.

### Stromal cells with distinct immunophenotypes show diverse transcriptome dynamics upon activation

Inflammation is known to trigger stromal cell activation globally. Based on the above data, the 3 subpopulations exhibited divergent characteristics, as well as distinct activation dynamics in the immobilization models. As inflammation in the above-mentioned immobilization models is obscure, we sought to identify transcriptomic profiling of the 3 subpopulations in their best-characterized model harboring diffuse inflammation. Stromal cells were isolated from samples obtain on d 3 of acute muscle injury and from control muscles, and were sorted based on the above-mentioned immunophenotypes. The sorting efficiency is presented in S2 Fig. Gene expression signatures of the stromal cell subpopulations were investigated using RNAseq analysis. The raw data and normalized gene counts are accessible from the on-line GEO database (GSE169411).

**Fig 4:**
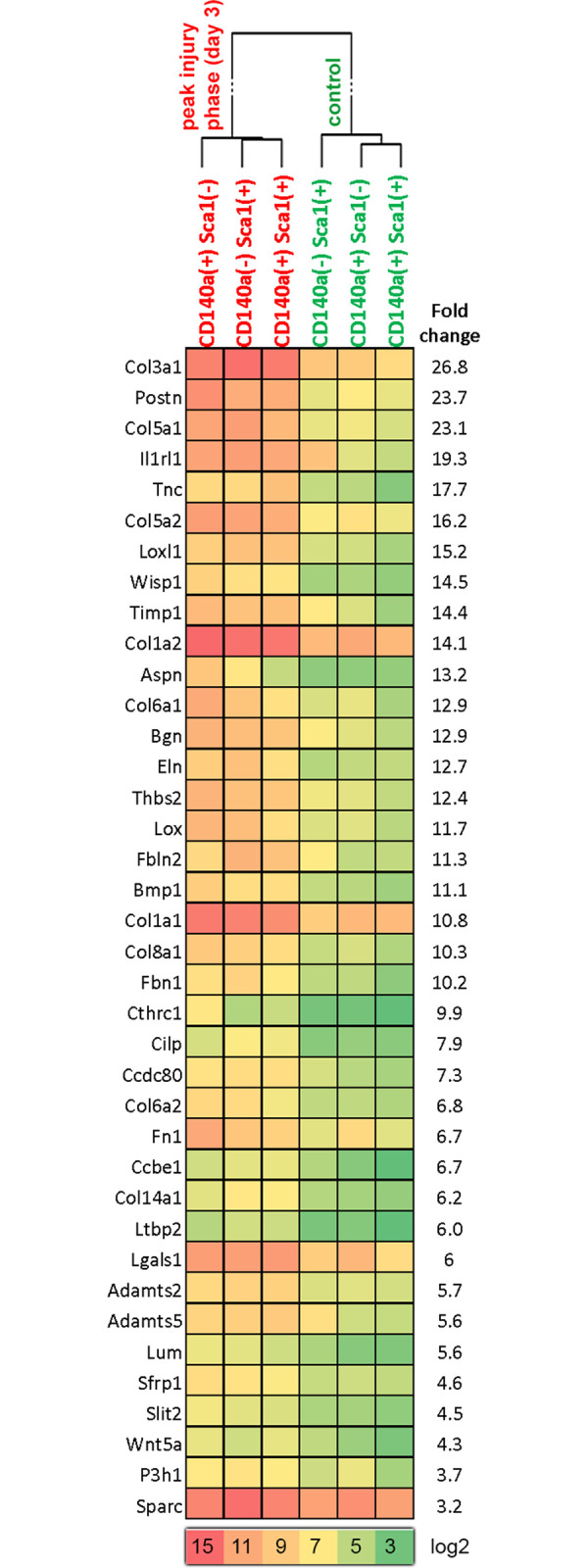
The common subset of upregulated ECM components that sharply differentiated (please see the dendrogram) the control samples from peak injury phase (d 3) are presented as a heat map (log2).

Initially, univariate class comparison (2-sample t-test with random variance model) was performed to compare the control and acute injury samples using a paired sample option, a significance cut-off of P < 0.01, and a fold change cut-off of ±3. This showed that there were 355 genes that exhibited significant alteration of expression, of which 219 were upregulated and 136 were downregulated (dendrogram is presented in S3 Fig and the gene table is provided in S1 Table). The upregulated gene list was annotated using the DAVID tool [28], showing 2 major annotation clusters with enrichment scores of 26.50 and 24.73, in which both contained genes of secreted ECM components (S2 Table). A majority of the collagens and other structural genes related to extracellular remodeling was prominent in the upregulated gene list. The dynamic heat-map clustering of the genes contained in this highest enrichment cluster are presented in Fig 4. A similar analysis of the significantly downregulated genes showed downregulation of angiogenesis and tissue homeostasis-related genes, indicating a relative dilution effect due to loss of tissue integrity.

**Fig 5:**
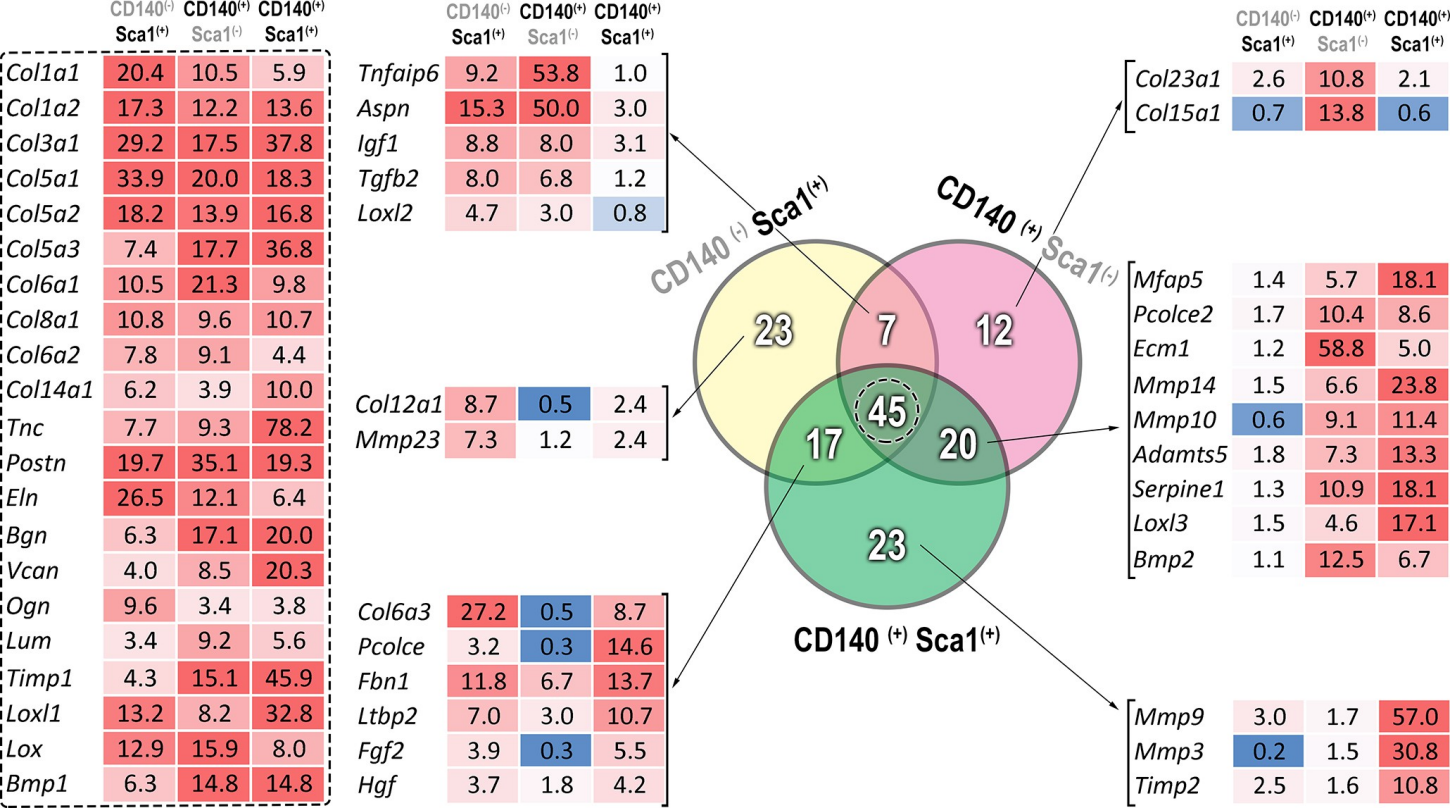
The matrisome gene ontology classification of relevant genes were extrapolated on a converged gene list converged via maximally expressing genes (≥75^th^ percentile) exhibiting highest fold changes of the investigated stromal cell populations. In total, 45 ECM structural proteins were commonly upregulated in all 3 stromal cell subpopulations (dashed box/left panel). Prominent transcripts with known function in ECM regulation, in which upregulation is observed, particularly in a single or shared cell subpopulations, are shown.

We further attempted to identify major transcripts that delineated the 3 subpopulations and their contribution to ECM. The 300 highest expressing genes exhibiting the highest variation (FC>3) in response to injury were unified. In all, 479 converged transcripts were annotated according to the matrisome gene ontology classification proposed by Naba et al. [29]. The selected matrisome components expressing the most striking commonality (45 transcripts) and discordance are presented in Fig 5 (the matrisome annotation table is presented in S3 Table). The findings show that the majority of the collagens were upregulated in a similar mode across all 3 subpopulations; however, the extent of upregulation varied for the ECM glycoproteins. Likewise, tenascin c and elastin exhibited the highest and the lowest upregulation, respectively, in the Sca1^(+)^/CD140^(+)^ subpopulation. A similar disproportionate upregulation was also prominent for the matrisome associated regulators, such as Timp1 and Bmp1, which exhibited the least upregulation in the Sca1^(+)^/CD140^(–)^ subpopulation. A disparate expression pattern was also observed for growth factors, where FAPs are known to support myogenesis through. Induction of IGF1 expression was limited in the Sca1^(+)^/CD140^(+)^ subpopulation. Likewise, the Sca1^(–)^/CD140^(+)^ subpopulation did not exhibit significant upregulation of FGF2 (*bFGF*) or HgF2.

## Discussion

Among the hallmarks of skeletal muscle degeneration, fibrosis is the most prominent one leading to progressive functional loss and deterioration. FAPs are a heterogeneous subset of skeletal muscle stromal cells that support injury repair and are the main cellular contributor to the development of fibrosis. The present study utilized the widely accepted immune phenotype of CD140a (PDGFRa) and/or Sca1 positivity, and exclusion of hematopoietic and endothelial markers [12, 27] to differentiate FAPs. This rare population of cells, are required for the physiological maintenance [30] and regeneration of skeletal muscle. TGFβ and TNFα [10] are the most well-known pro-inflammatory cytokines that regulate this cell population.

Tenotomy and denervation mimic the fundamental pathological features of chronic degeneration [31, 32] in which inflammatory processes are much more limited than in the case of acute injury evoked by phospholipases (cardiotoxin or notexin). Thus, immobilization models are valuable for the observation of the initiating events that drive the activation of FAPs in the absence of a prominent inflammatory background. The present study aimed to characterize the activation kinetics of stromal cell subpopulations using tenotomy and denervation models.

The present findings show that proliferative activation of FAPs along with immobilization is delayed to a greater degree than in the case of acute muscle injury. In the case of acute muscle injury infiltration of inflammatory cells rapidly trigger activation of FAPs, peaking on d 3 post injury [10]. Apparently, mononuclear cell proliferation following denervation peaks on d 10 to d 14 (2.2-fold) and exhibits a plateau following tenotomy on d 3 to d 7 (3-fold). Fibrosis is accompanying this increase in mononuclear cell number in tenotomy as shown by collagen deposition documented by Sirius red staining (Fig 1C) and verified by rtPCR (Fig 1E). These findings highlight the fact that the molecular events that trigger the proliferation of stromal cells following immobilization progress slowly and last longer than an inflammatory background. The kinetics and events resulting in this plateau effect are primarily due to variations arising from the nature of the models [18].

FAPs are known to consist of a heterogeneous population [33, 34]. The present findings show that tenotomy and denervation evoked a striking variance in activation of stromal cell subpopulations harboring different immunophenotypes. Acute injury induced significant increase of the CD140a^(+)^/Sca1^(+)^ subpopulation, as in the denervation model, but was very limited (6-fold) in the latter case; however, following tenotomy, the major subpopulation that exhibited increase was CD140a^(−)^/Sca1^(+)^. This subpopulation exhibited up to 3-fold increase from d 3 to d 7, which is in-line with the kinetics of total stromal cells (Fig 1), showing that the causative cellular events progress slowly but persistently.

Myofiber damage leads to rapid infiltration of inflammatory cells. The present findings show that almost 50% of the mononuclear cell population in muscle were of CD45^(+)^, CD11b^(+)^, and CD31^(+)^ cells on d 3 following acute injury; however, there was no direct fiber damage to induce such inflammatory activation in the cases of tenotomy and denervation. In control muscle only 10% of the mononuclear cells were CD45^(+)^, CD11b^(+)^, and CD31^(+)^, and this immunophenotype comprised no more that 15% in any of the investigated immobilization models (Fig 3). The investigated models of immobilization exhibited similar rates for this subpopulation (≥12.9% on d 7 post tenotomy and 15% on d 15 post denervation), along with the activation of diverse subpopulations of FAPs. The observed small increase in the inflammatory cell population is not sufficient to explain the diversity of the activated FAPs in the immobilized muscle. Here, we did not observe any significant variance within studied sub-populations of the control samples of hindlimb (gastrocnemius and soleus) and tibialis anterior muscles. However, CD140a^(+)^ Sca1^(+)^ exhibited highest variation within the controls. Further investigation is required to elucidate the number of resident FAPs within different muscle groups.

Non-physiological shortening of fibers of tenotomized muscle was suggested to cause partial segmental necrosis that initiates phagocytic cell activation [35]. Unlike in the case of tenotomy immobilization, denervated muscles protect their architecture. Earlier studies proposed that monocyte infiltration following MCP1 release from the axonal ends in the perisynaptic area was an initiator of inflammatory events following denervation [36]. Similar findings were observed in an SOD1 knock-out familial ALS model [37]; however, none of the above-mentioned mechanisms adequately explain the diversity and kinetic variance of the activated subpopulations observed in the present study’s 2 immobilization models.

In the light of the above-mentioned studies, the present findings indicate that there may be a minute (if any) local or very limited inflammatory response in the studied immobilization models. In an earlier study we postulated that there might be a pathomechanical scenario in which abrupt shortening and an increase in mid-belly diameter of tenotomized muscle might induce activation of ECM-resident TGFβ [18]. The same could also be possible in the case of denervation, in which supra-physiological reduction of the fiber diameter secondary to atrophy could trigger TGFβ activation. Likewise, non-physiological kinetic alterations of the ECM structure might activate ECM modulators (likewise MMPs and TIMPs), which may directly or indirectly contribute to the activation of FAPs [15, 38].

The present study also comparatively investigated the transcriptomes of the studied stromal cell subpopulations, so as to identify common and divergent events in response to inflammation, as well as to compare their resting-state gene expression profiles. In the acute injury model d 3 was selected for comparative analysis of stromal subpopulations and molecular activation signatures based on the fact that d 3 is the peak for activation [33, 39, 40]. Due to the scarcity of the cells, it was not possible to conduct a comparative transcriptome analysis of the subpopulations in immobilization models. The signaling pathways acting on the studied models are diverse and transcriptional profiles of these cells are also expected to be different depending on the model. Based on this limitation of this study, we conducted a descriptive phenotypic transcriptome analysis. The common subset of genes that were upregulated following injury included several structural ECM components, such as collagens and ECM remodelers, including *Bmp1*, *Sfrp1*, *Sfrp2*, periostin (*Postn*), and *Mmp19* (S1 Table and Fig 4). *Wnt5a* is a striking member of this gene list and supports previous findings of Wnt signaling in FAP biology [41].

Furthermore, the present study attempted to identify specific subpopulation-type sets of transcripts or profiles that were upregulated upon activation. Most of the upregulated transcripts in the Sca1^(+)^/CD140^(−)^ and Sca1^(−)^/CD140^(+)^ subpopulations exhibited significant overlap. Among these transcripts, collagens and several ECM components were the most prominent; however, striking upregulation of Tnfaip6 and Aspn differentiated the Sca1^(−)^/CD140^(+)^ subpopulation from the others. The Sca1^(+)^/CD140^(+)^ subpopulation exhibited the highest upregulation of Mmp3, Mmp9, and Timp2; however, additional research is needed to validate these proteins as potential pathway predictors or biomarkers.

One critical dilemma related to Sca1 antigen is its absence in humans; therefore, the present study attempted to identify potential cell surface proteins that correlated to Sca1 expression. Tmem106a, Cstb, Slc2a9, and Serpinb6a exhibited a >0.7 correlation to Sca1 expression in the whole data set; however, the validity of these potential markers needs to be further elucidated. A number of biomarkers were previously reported to correlate with FAP phenotype and we further evaluated the expression of some previously reported FAP-related biomarkers in our data-set.

Firstly, the present study focused on highly expressed genes that play a role in tissue remodeling and ECM architecture, and are activated via injury in all 3 studied stromal subpopulations. Although all 3 of investigated stromal cell subpopulations share a somewhat similar expression profile of ECM components, interestingly only the Sca1^(−)^/CD140^(+)^ subpopulation exhibited striking proliferation in response to injury (Fig 2). This observation shows that activation towards proliferation and induction of pro-fibrogenic tissue remodeling are 2 distinct events triggered by common mediators of pro-inflammatory signaling. Interestingly, the CD140a^(+)^/Sca1^(−)^ subpopulation exhibited the greatest level of LepR (leptin receptor) positivity and it’s expression was uniquely enhanced by injury (S4 Table). It was previously reported that LepR^(+)^CD140a^(+)^CD45^(−)^ mesenchymal stromal cells are the primary cellular source of myelofibrosis in bone marrow. These cells were identified as the supporting cellular components for AML and responsible for the fatty degeneration of bone marrow [42, 43]. Although the LepR^(+)^ phenotype is not investigated in skeletal muscle (yet), CD140a^(+)^/Sca1^(−)^ is likely to be a corresponding subpopulation accompanying fatty degeneration secondary to metabolic ECM changes in patients with diabetes and obesity. In contrast, CD140a^(−)^Sca1^(+)^ is a much more heterogeneous subpopulation, as Sca1 is a ubiquitous marker in mice. Even myoblasts are known to be Sca1 positive; thus, in order to confirm the exclusion of any myoblasts, we cultured CD140a^(−)^/Sca1^(+)^ FAPs and induced adipogenic and myogenic differentiation. Effective adipogenic differentiation was easily observed, whereas myotube formation was absent (S4 Fig). The absence of any myoblast-specific gene expression based on our transcriptome studies supports the exclusion of myoblasts.

As earlier studies proposed a number of differentiating and specific markers for various FAP subpopulations, we looked for the presence of such markers in the present study’s data. Wisp1 was defined as a proliferation-enhancing factor for FAPs and was reported to diminish with age, limiting muscle regenerative capacity [44]. Wisp1 upregulation (almost 2-fold) was partially evident following injury in all stromal cell subpopulations investigated in the present study. Vcam1 and/or Tek expression was also suggested to be upregulated in an inflammatory environment [33]. Apparently, induction of Wisp1, LepR, and Vcam1 expression was only valid for the CD140a^(+)^/Sca1^(−)^ subpopulation (S4 Table), signifying the highly heterogeneous nature of FAPs and indicating that there are divergent mechanisms controlling the proliferative activation and secretory (ECM) response to environmental stimuli. The present study’s findings further exclude inflammation as a *sine-qua-non* for fibrosis.

## Conclusion

FAPs are a heterogeneous population that can exhibit a variety of kinetics in the inflammatory environment following acute tissue injury. To the best of our knowledge the present study is the first to show that inflammation may not be a pre-requisite for FAP activation following immobilization. The delayed proliferation kinetics observed in response to immobilization indicate that signals other than pro-inflammatory cytokines may trigger activation of FAPs, leading to irreversible architectural changes in the stroma. Diverse cellular subpopulations contribute to distinct aspects (ECM components) of skeletal muscle tissue in the case of remodeling. FAPs in skeletal muscle and stromal cells of other organs may have similar phenotypes and functions.

## Supporting information

S1 Fig Stromal cells were enumerated based on relevant immunophenotypes (CD140a(+) Sca1(-), CD140a(-) Sca1(+) and CD140a(+)Sca1(+)) and BrDU positivity. Typical flowcytometry panels from acute injury (day3), tenotomy (day 7) and denervation (day10) are shown in the upper panel (n = 10). BrdU positivity of each stromal subpopulation is calculated and presented in pie charts in lower panel. Diminutive amount of BrdU positivity was observed in the control samples and the highest proliferation in stromal cells was observed following acute injury. Stromal cells from the immobilization models exhibited limited BrdU positivity (n = 10 for each group).
(TIF)

S2 Fig Stromal cells isolated from the control muscles and the 3rd day samples following acute injury. Cells were sorted based on relevant immunophenotypes. Sorting efficiencies for each sub-population was assessed using flow cytometry. Lowest enrichment was observed in CD140a(+)Sca1(+) population in control samples (80%).
(TIF)

S3 Fig Unsupervised distribution dendrogram of samples (all transcripts) are shown in A. Distribution of samples using significant genes (S1 Table) are presented in B.
(TIF)

S4 Fig Stromal cells were sorted based on CD140a(-) Sca1(+) immunophenotype and were cultured to confluence (upper panel). Upon reaching confluence, cells were subjected to adipogenic or myogenic differentiation and observed up to 10 days. No myofibers was observed along the observation period. Lipid accumulation could be observed in some of the cells (mid panel). On the contrary, induction of adipogenic differentiation could be robustly induced in the majority of the cells (lower panel).
(TIF)

S1 Table A paired t-test was performed to compare the gene expression profiles of stromal cells in control and acute injury conditions. This revealed 355 genes that exhibited significant alteration of expression where 219 were upregulated and 136 were downregulated. Genes were filtered with a significance cut-off of p<0.05 and a fold change cut-off of ±3.
(XLSX)

S2 Table Upregulated gene list was annotated using the DAVID tool that revealed two major annotation clusters with enrichment scores of 26.50 and 24.73, both containing genes of secreted extracellular matrix components. (XLSX)

S3 Table Unified list of the highest expressing (top 300) genes exhibiting the highest variation (FC>3) in response to injury in the three studied cell subpopulations. This signature was annotated using the matrisome gene ontology classification presented in Fig 5.
(XLSX)

S4 Table Expression of previously identified markers attributed to FAPs are documented in the investigated subpopulations. (XLSX)
