# Supplementary figures and images for: Pro-fibrogenic and adipogenic aspects of chronic muscle degeneration are contributed by distinct stromal cell subpopulations
Source: PLoS One. 2023 Jul 18;18(7):e0288800. doi: 10.1371/journal.pone.0288800 (PMC10353787; doi:10.1371/journal.pone.0288800)

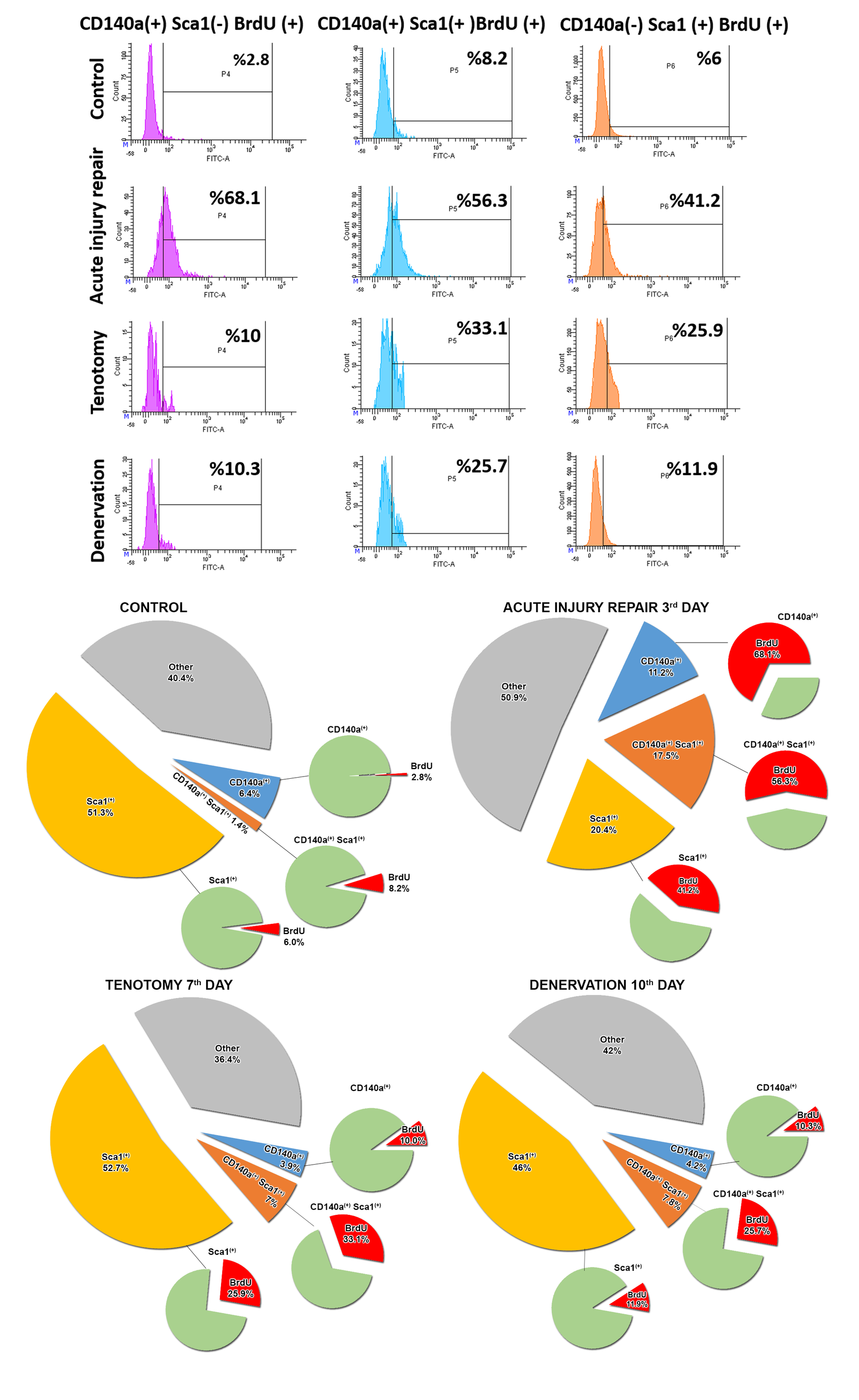

Supplement: S1 Fig — Typical flowcytometry panels from acute injury (day3), tenotomy (day 7) and denervation (day10) are shown in the upper panel (n = 10). BrdU positivity of each stromal subpopulation is calculated and presented in pie charts in lower panel. Diminutive amount of BrdU positivity was observed in the control samples and the highest proliferation in stromal cells was observed following acute injury. Stromal cells from the immobilization models exhibited limited BrdU positivity (n = 10 for each group). (TIF) [file pone.0288800.s001.tif]

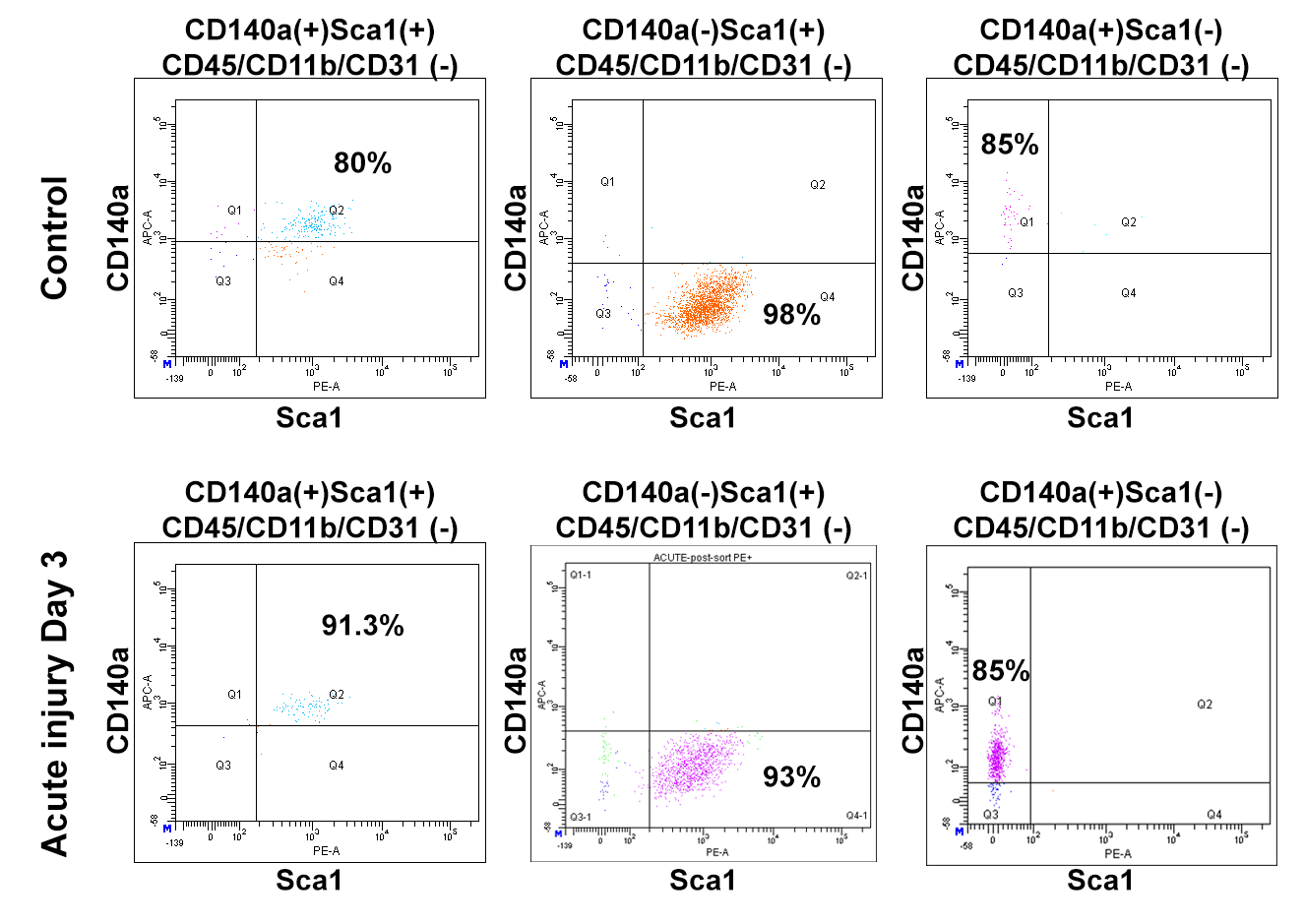

Supplement: S2 Fig — Cells were sorted based on relevant immunophenotypes. Sorting efficiencies for each sub-population was assessed using flow cytometry. Lowest enrichment was observed in CD140a(+)Sca1(+) population in control samples (80%). (TIF) [file pone.0288800.s002.tif]

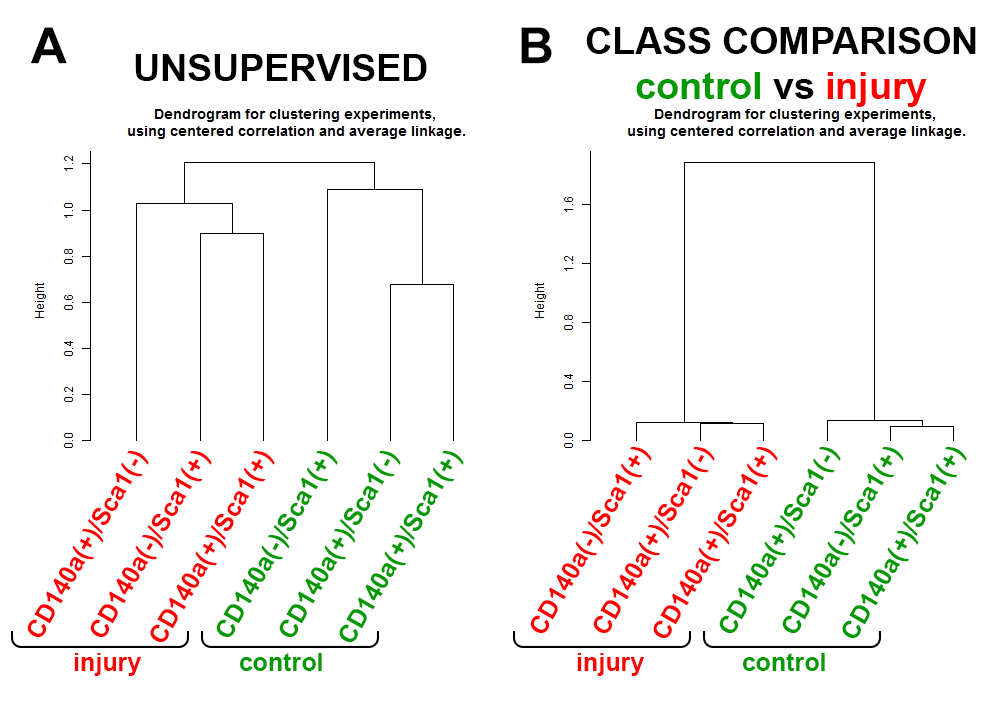

Supplement: S3 Fig — Unsupervised distribution dendrogram of samples (all transcripts) are shown in A. Distribution of samples using significant genes (S1 Table) are presented in B. (TIF) [file pone.0288800.s003.tif]

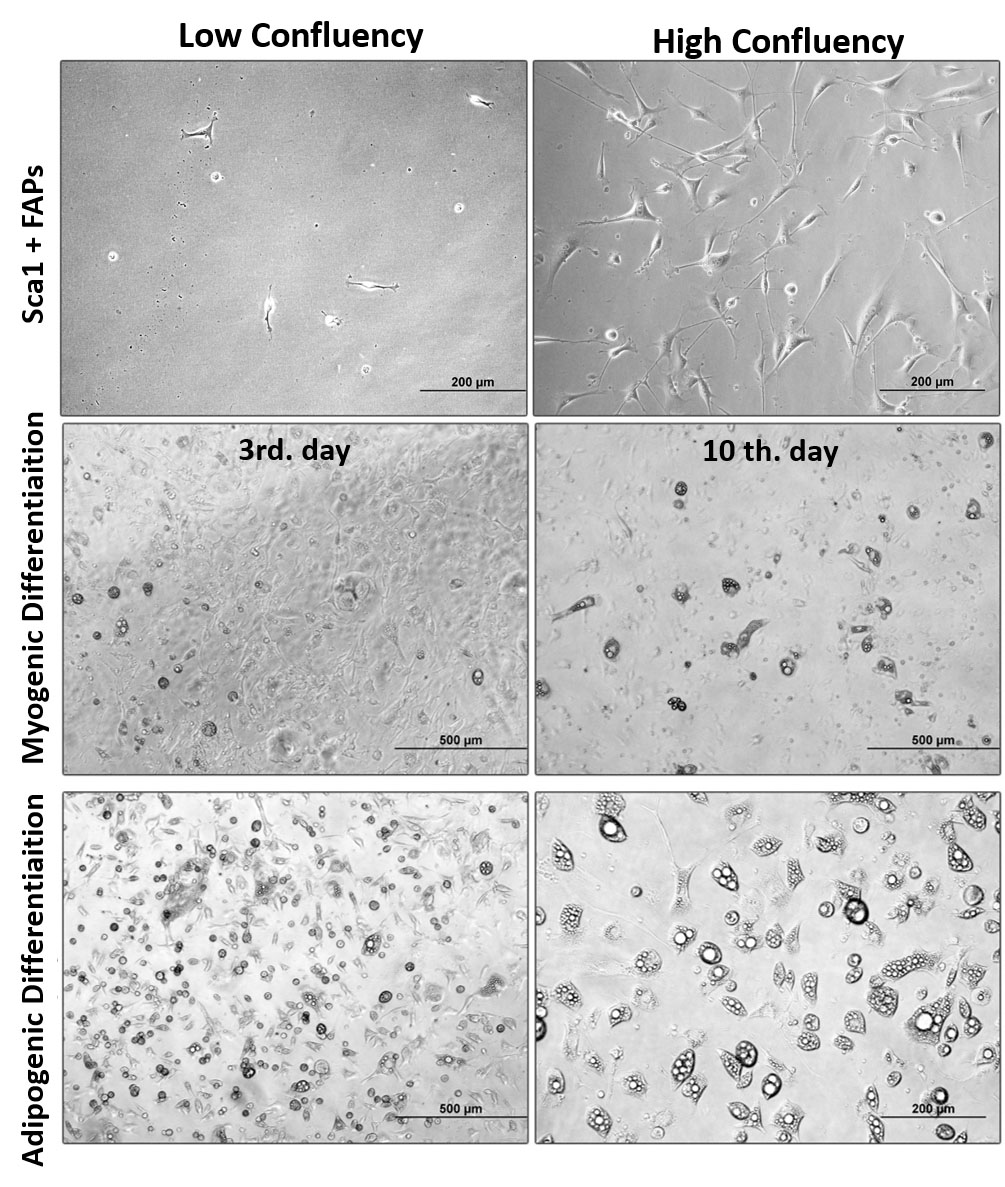

Supplement: S4 Fig — Stromal cells were sorted based on CD140a(-) Sca1(+) immunophenotype and were cultured to confluence (upper panel). Upon reaching confluence, cells were subjected to adipogenic or myogenic differentiation and observed up to 10 days. No myofibers was observed along the observation period. Lipid accumulation could be observed in some of the cells (mid panel). On the contrary, induction of adipogenic differentiation could be robustly induced in the majority of the cells (lower panel). (TIF) [file pone.0288800.s004.tif]
